# Laparoscopic versus open hemihepatectomy: comprehensive comparison of complications and costs at 90 days using a propensity method

**DOI:** 10.1007/s13304-020-00854-y

**Published:** 2020-07-30

**Authors:** Francisco Riquelme, César Muñoz, Fabio Ausania, Amelia J. Hessheimer, Ferrán Torres, David Calatayud, Raffaele Sandomenico, Rocío García Pérez, Joana Ferrer, José Fuster, Juan Carlos García-Valdecasas, Constantino Fondevila

**Affiliations:** 1 Hepatopancreatobiliary Surgery and Transplantation, General and Digestive Surgery Service, Hospital Clínic, IDIBAPS, CIBERehd, University of Barcelona, Barcelona, Spain; 2 Medical Statistics Core Facility, Faculty of Medicine, IDIBAPS, Hospital Clínic Barcelona, Barcelona, Spain, and Biostatistics Unit, Universidad Autónoma de Barcelona, Barcelona, Spain; 3 Department of General and Digestive Surgery, Hospital Clínic, University of Barcelona, C/ Villarroel 170, 08036 Barcelona, Spain

**Keywords:** Hemihepatectomy, Laparoscopy, Major liver resection, Comprehensive complication index

## Abstract

Laparoscopic hemihepatectomy (LHH) may offer advantages over open hemihepatectomy (OHH) in blood loss, recovery, and hospital stay. The aim of this study is to evaluate our recent experience performing hemihepatectomy and compare complications and costs up to 90 days following laparoscopic versus open procedures. Retrospective evaluation of patients undergoing hemihepatectomy at our center 01/2010–12/2018 was performed. Patient, tumor, and surgical characteristics; 90-day complications; and costs were analyzed. Inverse probability of treatment weighting (IPTW) was used to balance covariates. A total of 141 hemihepatectomies were included: 96 OHH and 45 LHH. While operative times were longer for LHH, blood loss and transfusions were less. At 90 days, there were similar rates of liver-specific and surgical complications but fewer medical complications following LHH. Medical complications that arose with greater frequency following OHH were primarily pulmonary complications and urinary and central venous catheter infections. Complications at 90 days were lower following LHH (Clavien–Dindo grade ≥ III OHH 23%, LHH 11%, *p* = 0.130; Comprehensive Complication Index OHH 20.0 ± 16.1, LHH 10.9 ± 14.2, *p* = 0.001). While operating costs were higher, costs for hospital stay and readmissions were lower with LHH. Patients undergoing LHH experience a significant reduction in postoperative medical complications and costs, resulting in 90-day cost equity compared with OHH.

## Introduction

The laparoscopic approach to abdominal interventions better preserves the integrity and function of the abdominal wall and limits exposure and manipulation of abdominal viscera, not only leading to improved cosmesis but also reduced intraabdominal adhesions and wound and wall complications. The loss of manual tissue manipulation and control is compensated, at least to some degree, by magnified and direct visualization. In the field of hepatic surgery, the results of clinical retrospective studies, meta-analyses, and one randomized trial indicate that the aforementioned advantages may translate into less perioperative blood loss and improved recovery, with decreased postoperative complications and hospital stay [1–4]. For these reasons, the laparoscopic approach is now considered standard-of-care for the resection of lesions in the left lateral and anterior hepatic segments [5].

In contrast with the aforementioned minor liver resections, laparoscopic hemihepatectomy (LHH) is still considered an advanced technique to only be carried out by experienced surgeons. While transection may proceed across hepatic anatomical planes, these planes remain intraparenchymal, highly vascularized, and not recognized by standard traction–countertraction maneuvers. As well, vascular and biliary structures to one side of the liver have to be ligated while preserving structures on the contralateral side, and the liver has to be separated to a certain extent from the inferior vena cava, intraoperative injury to which can be devastating. Accordingly, a learning curve of at least 55 cases for right hemihepatectomy after prior experience with minor laparoscopic liver resections has been suggested [5, 6].

Aside from increased complexity, cost associated with LHH can also be increased, in particular related to the use of more expensive surgical instruments and devices and longer operative times versus open hemihepatectomy (OHH). Cost analyses have determined that operative costs may be significantly higher for LHH than for OHH, though the increase in upfront cost may be offset, at least in part, by improved and less costly postoperative recovery and hospital stay [7–10].

The laparoscopic approach to hepatic resection was introduced at our center in 2005, and we currently perform approximately 75% of our hepatectomies laparoscopically. Based on the results of previous studies, we hypothesized that fewer complications arise after LHH and this difference may translate into not only a shorter and less costly postoperative stay but also fewer readmissions and ongoing complications through the end of the third postoperative month, whereby the great majority of complications following major hepatectomy has occurred [11]. The aim of the present study is, therefore, to comprehensively evaluate the recent years of our experience performing hemihepatectomy and to compare cumulative complications and costs measured up to 90 days following laparoscopic versus open procedures.

## Patients and methods

### Study design

This study is a retrospective evaluation of patients undergoing right or left hemihepatectomy at our center between January 2010 and December 2018. Exclusion criteria included extended hepatectomy, additional biliary or vascular resection, previous portal vein embolization, previous hepatectomy (including two-stage hepatectomy), emergency hepatectomy, living donor hepatectomy, and synchronous multiorgan resection (including bowel resection). Approval for the study was obtained from our Institution’s Committee on Ethics in Medical Research prior to initiating data analysis (study number HCB/2018/1179), which waived the need for written consent.

### Preoperative evaluation

Preoperatively, all patients underwent routine laboratory tests and contrast-enhanced multislice abdominal computed tomography (CT) and and/or gadolinium-enhanced hepatic magnetic resonance imaging (MRI) and were discussed in multidisciplinary tumor board. For colorectal liver metastases, preoperative chemotherapy was indicated in cases of initially unresectable disease until conversion to resectability, borderline resectable disease, and initially resectable disease with synchronous presentation. Initial resection of the primary tumor was performed in cases with synchronous presentation.

### Surgical procedures

All surgeons performing LHH had previously performed a minimum of 30 laparoscopic liver resections, and all surgeons performing both LHH and OHH had extensive experience performing open hepatic surgery.

#### Laparoscopic hemihepatectomy

The patient was placed with legs spread and in reverse Trendelenburg. Five or six ports were used, with the 12-mm camera port placed along the anterior axillary line. Pneumoperitoneum was maintained 12–14 mmHg. Following mobilization of the right or left liver from its ligamentous attachments, intraoperative ultrasound was performed to confirm the number and size of the lesion(s). Preparation for intermittent Pringle maneuver was performed by encircling the hepatic hilum with a tourniquet, which was exteriorized through a thoracostomy tube inserted through the abdominal wall above the pubic symphysis. An extrahepatic intraglissonian pedicle approach was used to dissect and encircle the ipsilateral hepatic artery and portal vein. For left LHH, the left lobe was pulled upward, and Arantius’ ligament approach was used to encircle the left hepatic vein [12]. For right LHH, retrohepatic veins and the hepatocaval ligament were divided between titanium or Hem-*o*-lok® clips, followed by dissection and encircling of the right hepatic vein to perform liver hanging maneuver. Ischemic delimitation of the transection plane was achieved after pedicle clamping, and liver transection was performed using a combination of LigaSure™ (Medtronic, Minneapolis, Minnesota, USA) and CUSA® Excel (Valleylab, Boulder, Colorado, USA). Vascular staplers were used to transect both the hepatic pedicles at the level of the hilar plate and hepatic veins and the end of parenchymal transection. The surgical specimen was placed in an Endo Catch™ Specimen Retrieval Pouch (Medtronic, Minneapolis, Minnesota, USA) and removed through a Pfannenstiel incision. The incision was then closed, intraabdominal hemostasis was checked, and an abdominal drain was placed.

#### Hybrid hemihepatectomy

The hybrid technique was defined as a planned procedure that started with liver mobilization and isolation of hepatic pedicle through laparoscopic approach. Thereafter, parenchymal transection was performed through a 6–8 cm upper midline laparotomy, which was also used for specimen extraction.

#### Open hemihepatectomy

Open approach was performed using a “J”-shaped or right subcostal incision with subxiphoid extension. After exploration of the abdominal cavity and hemiliver mobilization, intraoperative ultrasound was performed. The hepatic hilum was encircled with a tourniquet to allow subsequent intermittent Pringle maneuver. The ipsilateral hepatic artery and portal vein were then dissected and clamped in the hepatic hilum, delimiting the transection plane. CUSA® was used for parenchymal transection. The hemiliver pedicle was sectioned between sutures and the suprahepatic vein using a vascular stapling device. An abdominal drain was routinely placed.

### Definition of variables and outcomes

The main objective of the study was to assess clinical outcomes and costs associated with LHH and OHH up to 90 days postoperatively. Cases of LHH that resulted in conversion to open surgery were not censored and were included among the LHH group for all analyses based on intention-to-treat. The following patient-related variables were recorded: age, sex, body mass index (BMI), American Society of Anesthesiologists (ASA) class, and previous abdominal surgery (open or laparoscopic surgery or percutaneous radiofrequency ablation). Co-morbid conditions were quantified according to the age-adjusted Charlson comorbidity index, which was calculated excluding liver tumor variables [13]. Liver-specific variables that were recorded included principle diagnosis and history of liver disease. Tumor-specific variables included the number, size, extent, and distribution of lesions and history of preoperative chemotherapy. Intraoperative variables included associated procedures (e.g., non-anatomical resection or radiofrequency ablation in the contralateral hemiliver), the use and duration of intermittent Pringle maneuver, operative time, blood loss, and reasons for conversion to open surgery in the LHH group. Surgical pathology was evaluated for tumor size and margins, with resection margins distancing  ≤ 1 mm from tumor cells classified as “R1”. Postoperative outcomes that were recorded included the need for blood transfusion, postoperative intensive care stay, postoperative hospital stay, complications, re-interventions, unplanned readmissions, and mortality. All complications occurring up to 90 days postoperatively were recorded and graded according to the Clavien–Dindo [14] and Comprehensive Complication Index (CCI) systems, the latter of which computes all the complications a patient experiences in an index ranging from 0 to 100 [15]. Postoperative complications were categorized as surgical, medical, or liver specific. Liver-specific complications were classified according to the International Study Group for Liver Surgery (ISGLS) criteria. Bile leak was defined as a drain bilirubin concentration > 3-times higher than the serum bilirubin concentration at or beyond the third postoperative day and graded as A—requiring no change in patient’s clinical management, B—requiring therapeutic intervention but manageable without surgical re-intervention, or C—requiring surgical re-intervention [16]. Post-hepatectomy liver failure (PHLF) was defined as increased international normalized ratio (INR) and hyperbilirubinemia at or beyond the fifth postoperative day and graded as A—analytical deterioration not requiring any change in management, B—deviation from regular postoperative clinical pathway managed without invasive treatment, or C—PHLF requiring an invasive procedure [17]. Postoperative ascites was defined as drain fluid output > 10 mL/kg/day at or beyond the third postoperative day [18]. In addition to the hospital electronic medical record, the regional shared electronic medical record was checked for readmissions in other centers.

### Costs

Intraoperative, postoperative, and readmission costs were obtained, including variable and fixed costs that were charged in each case. Intraoperative costs included consumables (anesthetic drugs, transfusions, surgical instrumentation, and devices) and operating room and staff costs. Postoperative costs included pharmacy, transfusions, ICU and/or floor beds, studies and procedures (radiological, endoscopic, and/or surgical), and other miscellaneous expenditures (laboratory, nursing, and other professional care). Readmission expenses including re-intervention were grouped under total readmission costs. Taking into account the study inclusion period (2010–2018), costs were adjusted according to the mean annual inflation rate: 2010 + 1.8%, 2011 + 3.2, 2012 + 2.44, 2013 + 1.42, 2014 − 0.15, 2015 − 0.5, 2016 − 0.2, 2017 + 2.97, 2018 + 1.67 [19].

### Data and statistical analysis

Categorical variables are described as frequencies and percentages and continuous variables as mean ± standard deviation or median (25–75% interquartile range). The propensity score method, which simulates the effects of a randomized trial for observational data, was used to estimate study outcomes. Inverse probability of treatment weighting (IPTW) of the propensity scores was used to create a pseudo-population in which study groups were balanced across covariates using data blinded to outcomes. Weights were derived using logistic regression to estimate average treatment effects in treated patients and stabilized by treatment prevalence. The following covariates were included in the propensity models: age, sex, ASA classification, BMI, age-adjusted Charlson comorbidity index, previous abdominal surgery, previous radiofrequency ablation, cirrhosis, indication for surgery, number of liver lesions, diameter of the largest lesion, number of affected liver segments, unilobar versus bilobar liver disease, preoperative chemotherapy and number of cycles, and planned additional procedure. Categorical variables were compared using the Chi-square test and continuous variables using ANOVA with rank-transformed data for both raw and IPTW-adjusted analyses. Covariate balance was assessed using the standardized difference, which is the difference between groups divided by the pooled standard deviation. Statistical significance was defined as *p* < 0.05. Statistical analyses were performed using SAS 9.4 (SAS Institute, Inc., Cary, North Carolina, USA).

## Results

Between 01/2010 and 12/2018, 838 liver resections were performed at our center. Based on inclusion–exclusion criteria, 141 were included for analysis: 96 OHH and 45 LHH (70% and 64% right hemihepatectomies, respectively).

### Patient demographics and tumor and surgical characteristics

Baseline patient demographical information and tumor and surgical characteristics are listed in Table 1. The average patient in this study was around 65 years of age and overweight (BMI 25–26), with an ASA score of II. Men and women were equally represented among included patients. The indication for hemihepatectomy was malignant tumor in 90% of cases (OHH: 71% metastases, 22% primary liver tumors; LHH: 76% metastases, 9% primary liver tumors); consequentially, the majority of patients had a history of preoperative chemotherapy. In terms of tumor characteristics, the median number was two lesions affecting ≥ 2 liver segments, and the median size of the largest lesion was approximately 3 cm. Approximately a quarter of patients presented bilobar disease that required an associated procedure in addition to hemihepatectomy. On raw analysis, a greater percentage of patients in the LHH group had previously received chemotherapy and undergone laparoscopic abdominal procedures as opposed to open ones. Following IPTW adjustments, covariates were balanced between the two groups, with all *p* values < 0.05 and standardized differences < 0.25.

**Table 1:** Patient, tumor, and surgical characteristics used for the propensity model

	Raw analysis				IPTW analysis			
	OHH (n = 96)	LHH (n = 45)	*p* value	Standardized difference	OHH	LHH	*p* value	Standardized difference
Age (years)	65 [57–72]	65 [55–69]	0.476	− 0.120	65 [57–72]	67 [57–69]	0.889	0.028
Sex female	38 (40%)	23 (51%)	0.197	− 0.233	42 (45%)	16 (47%)	0.872	− 0.032
Body mass index	26 [23–28]	25 [23–28]	0.329	− 0.177	26 [23–27]	25 [23–28]	0.461	− 0.150
Age-adjusted Charlson comorbidity index	2 [1–4]	2 [1–3]	0.406	− 0.153	2 [2, 3]	2 [1–3]	0.669	− 0.087
ASA class								
I	4 (4%)	4 (9%)	0.258	0.192	4 (4%)	2 (5%)	0.872	0.032
II	71 (74%)	34 (76%)	0.839	0.037	70 (76%)	27 (77%)	0.885	0.029
III	21 (22%)	7 (16%)	0.380	− 0.163	19 (20%)	6 (18%)	0.810	− 0.048
Previous abdominal procedure	81 (84%)	38 (84%)	0.991	0.002	80 (86%)	30 (87%)	0.866	0.034
Radiofrequency ablation	3 (3%)	0	0.230	− 0.254	2 (2%)	0 (0%)	0.378	− 0.212
Open surgery	42 (44%)	9 (20%)	**0.006**	**− 0.527**	37 (39%)	10 (29%)	0.276	− 0.221
Laparoscopic surgery	39 (41%)	29 (64%)	**0.008**	**0.491**	43 (46%)	20 (58%)	0.244	0.233
Cirrhosis	3 (3%)	2 (4%)	0.692	0.069	3 (3%)	1 (3%)	0.973	0.007
Malignant disease	89 (93%)	38 (84%)	0.126	0.262	85 (91%)	30 (87%)	0.455	0.142
Preoperative chemotherapy	44 (46%)	29 (64%)	**0.039**	**0.381**	49 (52%)	21 (61%)	0.374	0.178
Multiple lesions	49 (51%)	26 (58%)	0.454	0.136	52 (56%)	19 (55%)	0.890	− 0.027
Number of lesions	2 [1–4]	2 [1–3]	0.903	0.023	2 [1–4]	2 [1–4]	0.593	0.109
Number of affected segments	2 [1–3]	2 [2, 3]	0.587	0.100	2 [2, 3]	3 [2, 3]	0.643	0.095
Maximum diameter of lesions (mm)	33 [22–55]	27 [15–45]	0.114	− 0.283	33 [22–55]	32 [15–45]	0.436	− 0.155
Bilobar disease	26 (27%)	11 (24%)	0.739	− 0.060	23 (25%)	10 (29%)	0.683	0.080
Planned associated procedure	30 (31%)	10 (22%)	0.267	− 0.205	27 (29%)	9 (26%)	0.798	− 0.051

Descriptive statistics are frequencies (%) for categorical variables and median [25–75% interquartile range]. Bold marked figures are for *p* < 0.05

*ASA* American Society of Anesthesiologists, *IPTW* inverse probability of treatment weighting, *LHH* laparoscopic hemihepatectomy, *OHH* open hemihepatectomy

### Operative characteristics and intraoperative outcomes

Table 2 lists variables associated with the surgical interventions and intraoperative outcomes. Rates of any associated intraoperative procedure were similar between the two groups (29% OHH, 26% LHH, *p* = 0.798). However, when analyzing associated procedures individually, more patients in the LHH group underwent concomitant radiofrequency ablation (2% OHH, 14% LHH), while more patients in the OHH group underwent concomitant non-anatomical resection in the remnant hemiliver (24% OHH, 12% LHH, *p* = 0.010). As well, more patients in the LHH group were subjected to intermittent Pringle maneuver (44% OHH, 84% LHH, *p* < 0.001).

**Table 2:** Operative variables and outcomes

	Raw analysis			IPTW analysis		
	OHH (*n* = 96)	LHH (*n* = 45)	*p* value	OHH	LHH	*p* value
Right hepatectomy	67 (70%)	29 (64%)	0.526	72%	61%	0.237
Associated procedure (any)	30 (31%)	10 (22%)	0.268	29%	26%	0.798
Non-anatomical resection	24 (25%)	5 (11%)	**0.018**	24%	12%	**0.010**
Radiofrequency ablation	2 (2%)	5 (11%)		2%	14%	
Other	4 (4%)	0		3%	0	
Pringle maneuver	42 (46%)	38 (86%)	** < 0.001**	44%	84%	** < 0.001**
Pringle maneuver (min)	18 [15–37]	33 [20–45]	**0.028**	17 [15–36]	30 [15–40]	0.081
Conversion to open surgery^a^	–	6 (17%)	–	–	–	–
Bleeding	–	2 (6%)	–	–	–	–
Adhesions	–	3 (9%)	–	–	–	–
Technical failure^b^	–	1 (3%)	–	–	–	–
Operative time (min)	210 [180–255]	270 [225–340]	** < 0.001**	210 [180–255]	270 [225–335]	** < 0.001**
Blood loss (mL)	300 [200–500]	200 [100–300]	**0.012**	300 [150–500]	200 [50–300]	**0.018**
R1 resection	13 (14%)	5 (11%)	0.656	13 (14%)	4 (11%)	0.612

Descriptive statistics are frequencies (%) for categorical variables and median [25–75% interquartile range]

*IPTW* inverse probability of treatment weighting, *LHH* laparoscopic hemihepatectomy, *OHH* open hemihepatectomy

^a^Calculated as a percentage of pure laparoscopic cases (*n* = 35)

^b^Failure of the laparoscopic CUSA®

In the LHH group, approach was purely laparoscopic in 35 cases (78%) and hybrid in 10 (22%). All hybrid cases were performed during the first 3 years of the study period. Among pure laparoscopic cases, conversion to open surgery was performed in six (17%): three for right upper quadrant adhesions, two for bleeding, and one for technical failure of the laparoscopic CUSA® device.

Operative times were significantly longer by 1 h in the LHH group (210 [180–255] minutes OHH, 270 [225–335] minutes LHH, *p* < 0.001), but LHH intraoperative blood loss was less (300 [150–500] mL OHH, 200 [50–300] mL LHH, *p* = 0.018). There were no differences in rates of “R1” resection (tumor cells ≤ 1 mm from the transection margin) detected between the two groups: 14% OHH and 11% LHH, *p* = 0.612.

### Postoperative complications and outcomes

Postoperative complications and events are described in Table 3. Consequent to less intraoperative blood loss, fewer patients undergoing LHH received perioperative blood transfusions (15% OHH, 1% LHH, *p* = 0.024). As well, fewer complications occurred in the LHH group prior to hospital discharge (overall 60% OHH, 39% LHH, *p* = 0.039), and CCI measured up to discharge was also lower with LHH (16.5 ± 16.1 OHH, 9.7 ± 14.2, *p* = 0.009). These differences were reflected in a reduction in ICU and overall postoperative hospital stays for patients in the LHH vs. OHH groups by a median of 2 days for each (*p* < 0.001 in both cases).

**Table 3:** Postoperative complications and outcomes evaluated up to 90 days postoperatively

	Raw analysis			IPTW analysis		
	OHH (*n* = 96)	LHH (*n* = 45)	*p* value	OHH	LHH	*p* value
**At discharge**						
Overall complications	62 (65%)	19 (42%)	**0.012**	60%	39%	**0.039**
Clavien–Dindo I/II	46 (48%)	15 (33%)	0.103	44%	29%	0.127
Clavien–Dindo ≥ III	16 (17%)	4 (9%)	0.217	16%	10%	0.416
CCI^a^	18.0 ± 16.2	10.7 ± 18.3	**0.001**	16.5 ± 16.1	9.7 ± 14.2	**0.009**
Overall postoperative stay (days)	8 [711]	6 [5–7]	** < 0.001**	8 [7–10]	6 [5–7]	** < 0.001**
ICU stay (days)	3 [1–4]	1 [0–3]	** < 0.001**	3 [1–4]	1 [0–2]	** < 0.001**
Blood transfusion	17 (18%)	1 (2%)	**0.010**	15%	1%	**0.024**
PRBC units	0.36 ± 0.92	0.04 ± 0.30	**0.010**	0.32 ± 0.87	0.02 ± 0.18	**0.018**
**At 90 days**						
Liver-specific complications^b^	16 (17%)	6 (13%)	0.611	16%	12%	0.600
Bile leak (any)	9 (9%)	4 (9%)	0.926	9%	9%	0.948
Grade A	2 (2%)	1 (2%)	0.396	2%	1%	0.610
Grade B	5 (5%)	0		4%	0	
Grade C	2 (2%)	2 (4%)		3%	5%	
Ascites	5 (5%)	2 (4%)	0.846	5%	3%	0.685
Encephalopathy	5 (5%)	0	0.119	4%	0	0.211
ISGLS PHLF (any)	50 (52%)	13 (30%)	**0.010**	53%	34%	0.053
Grade A	41 (43%)	12 (27%)	0.062	45%	32%	0.213
Grade B	8 (8%)	1 (2%)		8%	2%	
Grade C	1 (1%)	0		1%	0	
General complications	54 (56%)	14 (31%)	**0.005**	53%	28%	**0.010**
Surgical complications	23 (24%)	5 (11%)	0.075	22%	12%	0.199
Deep surgical site infection	11 (12%)	3 (7%)	0.375	10%	6%	0.546
Intestinal obstruction	2 (2%)	1 (2%)	0.958	2%	4%	0.459
Hemorrahge/hematoma	8 (8%)	0	0.046	7%	0	0.105
Superficial surgical site infection	8 (8%)	1 (2%)	0.166	9%	2%	0.148
Medical complications	37 (39%)	9 (20%)	**0.029**	37%	16%	**0.022**
Arrhythmia	2 (2%)	0	0.330	2%	0	0.443
Pneumonia	11 (12%)	1 (2%)	0.067	10%	1%	0.084
Pleural effusion	6 (6%)	0	0.087	6%	0	0.134
Paralytic ileus	12 (13%)	7 (16%)	0.620	11%	14%	0.688
Urinary tract infection	6 (6%)	0	0.087	6%	0	0.140
CVC infection	8 (8%)	0	**0.046**	7%	0	0.103
Pulmonary embolism	2 (2%)	1 (2%)	0.958	3%	1%	0.477
Overall complications	71 (74%)	23 (51%)	**0.007**	69%	44%	**0.010**
Clavien–Dindo I/II	48 (50%)	18 (40%)	0.267	46%	33%	0.189
Clavien–Dindo ≥ III	23 (24%)	5 (11%)	0.075	23%	11%	0.130
CCI^a^	21.6 ± 16.1	12.7 ± 18.3	** < 0.001**	20.0 ± 16.1	10.9 ± 14.2	**0.001**
Re-interventions at 90 days	6 (6%)	2 (4%)	0.666	6%	5%	0.758
Readmissions at 90 days	17 (18%)	3 (7%)	0.080	17%	4%	0.055
Mortality at 90 days	0	1 (2%)	0.143	0	1%	0.340

Descriptive statistics are frequencies (%) for categorical variables and mean ± standard deviation or median [25–75% interquartile range]

*CCI *Comprehensive Complication Index, *ICU* intensive care unit, *IPTW* inverse probability of treatment weighting, *ISGLS* International Study Group of Liver Surgery, *PHLF* post-hepatectomy liver failure, *PRBC* packed red blood cell, *SD* standard deviation

^a^CCI for entire group; for only patients that developed complications, CCI at discharge was 27.0 ± 12.2 OHH and 25.2 ± 20.8 LHH and at 90 days 28.4 ± 12.0 OHH and 24.8 ± 18.8 LHH

^b^Does not include ISGLS PHLF Grade A

By 90 days after surgery, overall complication rates were 69% OHH and 44% LHH (*p* = 0.01). Accordingly, CCI at 90 days was also lower with LHH (20.0 ± 16.1 OHH, 10.9 ± 14.2 LHH, *p* = 0.001). Upon examination of the specific complications that arose during 90 days, liver-specific complications, including bile leak, ascites, encephalopathy, and PHLF requiring a change in patient management, occurred at similar rates following both OHH and LHH. Surgical complications also occurred at relatively similar rates, though there was a tendency toward more postoperative hemorrhage and superficial surgical site infection among OHH patients. Medical complications, on the other hand, occurred at higher rates in the OHH group when compared with LHH (37% OHH, 16% LHH, *p* = 0.022). Medical complications were primarily pulmonary complications, including pneumonias and pleural effusions, and complications associated with the use of indwelling catheters, such as urinary tract infections and bloodstream infections arising from central venous catheters.

Rates of surgical re-intervention during the first 90 days did not differ between the OHH and LHH groups (6% and 5%, respectively, *p* = 0.758). Six patients undergoing OHH were re-operated: two each for hemorrhage and bile leak during the index admission and another two for bowel obstruction on a subsequent readmission. Two patients undergoing LHH were re-operated laparoscopically for bile leak during the index admission.

There was a greater tendency toward readmissions among patients undergoing OHH as opposed to LHH (17% vs. 4%, respectively), though this difference did not reach the level statistical significance (*p* = 0.055). In terms of mortality, there was only one death prior to 90 days in the entire cohort: a patient that underwent LHH died of massive pulmonary embolism during the first postoperative week.

### Costs

Perioperative costs are illustrated in Table 4. All operating room costs, including consumable material and costs associated with OR use and staffing, were higher for LHH versus OHH by a median of approximately 2500€ per procedure (*p* < 0.001). In contrast, postoperative costs, in particular those related to ICU and floor stays and medical staffing and care, were lower for LHH versus OHH by a median of roughly 2000€ per patient (*p* < 0.001). Costs for readmissions in Table 4 are presented as the median for all patients included in each group; costs for only cases where readmissions occurred were OHH (17 readmissions) 2049€ [1229–2411] and LHH 3 readmissions 1396€ [1229–4489], *p* = 1.0. Considering that median readmission costs were also reduced by about 200€ among LHH patients (a difference that did not reach statistical significance, *p* = 0.067), the overall balance of 90-day costs associated with OHH and LHH was similar: 9936€ [7134–11,908] and 10,448€ [7703–14,240], respectively, *p* = 0.286.

**Table 4:** Cost analysis evaluated up to 90 days postoperatively

	Raw analysis			IPTW analysis			
	OHH (*n* = 96)	LHH (*n* = 45)	*p* value	OHH	LHH	% difference	*p* value
Intraoperative costs							
Consumables	1801 [1468–2404]	3374 [2898–3889]	** < 0.001**	1777 [1429–2387]	3253 [2958–3889]	+ 83	** < 0.001**
OR time	1408 [1167–1748]	1827 [1452–2224]	** < 0.001**	1386 [1161–1745]	1730 [1393–2225]	+ 29	** < 0.001**
OR staff	821 [637–1262]	1290 [953–1551]	** < 0.001**	788 [632–1318]	1264 [902–1548]	+ 60	** < 0.001**
Total	4309 [3584–5348]	7239 [5542–8244]	** < 0.001**	4339 [3541–5346]	6917 [5314–8363]	+ 59	** < 0.001**
Postoperative costs							
Pharmacy	184 [50–297]	100 [30–175]	0.067	163 [39–274]	100 [27–157]	− 40	0.114
Transfusions	90 ± 162	22 ± 111	** < 0.001**	80 ± 153	10 ± 67	− 88	**0.001**
Postoperative stay	3896 [2603–4972]	2070 [1648–3313]	** < 0.001**	3574 [1978–4805]	2046 [1560–3012]	− 43	** < 0.001**
ICU	2379 [1121–3311]	797 [0–2212]	** < 0.001**	2212 [0–3188]	761 [0–1592]	− 65	** < 0.001**
Surgical floor	1492 [1183–1872]	1111 [857–1533]	** < 0.001**	1444 [1182–1850]	1182 [887–1560]	− 18	**0.002**
Techniques	142 [67–339]	87 [32–166]	**0.008**	124 [53–302]	60 [31–177]	− 69	0.086
Miscellaneous^a^	707 [501–1117]	515 [302–632]	** < 0.001**	651 [455–1075]	515 [294–580]	− 21	**0.002**
Total	4990 [3751–6434]	2924 [2079–4449]	** < 0.001**	4817 [2956–6251]	2875 [2066–4109]	− 40	** < 0.001**
Total surgical admission costs	9916 [7766–12290]	10,448 [7703–13730]	0.270	9254 [7134–11908]	10,396 [7540–13730]	+ 12	0.195
Readmission costs	372 ± 1043	232 ± 1195	0.140	331 ± 944	110 ± 699	− 67	0.067
Total cost at 90 days	10,112 [7849–12433]	10,931 [8374–14240]	0.300	9936 [7134–11923]	10,448 [7703–14240]	+ 5	0.286

Values are reported in Euros (€) and presented as mean ± standard deviation or median [25–75% interquartile range]

^a^Laboratory tests and medical care during floor stay

## Discussion

In this study, we meticulously evaluated complications and costs associated with hemihepatectomy arising between index procedures up to 90 days postoperatively. The major findings were a relatively high rate of largely medical complications following OHH that were concomitantly associated with increased ICU and overall postoperative hospital stays as well as higher postoperative costs relative to LHH. Readmission costs for OHH also tended to be higher. In contrast, upfront intraoperative costs associated with OHH were lower, and overall costs for the open and laparoscopic approaches to hemihepatectomy were equivalent after 3 months of follow-up.

Previous studies following hemihepatectomy patients during their index hospitalization up to the end of the first postoperative month have also found relative cost equity when comparing open and laparoscopic procedures [8–10]. It has been determined for major surgical interventions, however, that limiting the period of observation to the end of the first postoperative month may overlook significant further morbidity, mortality, and costs, and a 90-day observation period appears to more accurately capture all relevant postoperative events [11, 20–24]. In the present study, additional complications occurring beyond discharge were not negligible, with approximately 10% more complications arising between discharge and 90 days among OHH patients and 5% more complications between discharge and 90 days among those undergoing LHH. Additional postoperative complications are not only relevant to patient satisfaction and quality of life but are also known to be a primary factor that serve to increase healthcare costs related to surgery [25, 26].

The overall rate of complications detected by 90 days in this study was ostensibly high: 69% for OHH and 44% for LHH. On the other hand, rates of moderate and severe complications requiring invasive therapy or management in intensive care (i.e., Clavien-Dindo III–V: 23% OHH and 11% LHH) were relatively low in comparison with other recent studies examining outcomes following hemihepatectomy [6–10]. Moreover, among patients developing complications, CCI, which provides a comprehensive view of all postoperative complications, with each graded according to its clinical impact (i.e., resources required to treat) remained below benchmark values in both groups, both at discharge (< 27.9) and at 3 months (< 32.6) [11].

Liver-specific complications were similar between the two groups in this study, though there was a tendency towards a higher rate of grade A PHLF (ongoing abnormal laboratory values requiring no change in clinical management) following OHH. Rates of surgical complications (intraabdominal abscess/fluid collection, mechanical intestinal obstruction, hemorrhage/hematoma, and superficial surgical site infection) were the same. However, rates of medical complications were significantly higher for OHH.

Medical complications not reaching the point of requiring invasive and/or intensive care treatment may have been under-detected and/or -reported in previous retrospective studies evaluating outcomes following hemihepatectomy [6, 7, 10, 27]. We exhaustively evaluated all postoperative events, including delay in return of normal bowel function, which was surprisingly not improved among patients undergoing LHH. Manipulation of the bowel in both LHH and even OHH remains limited with respect to other intraabdominal procedures, yet hemihepatectomy can significantly impact bowel function based on the aggressive nature of the procedure itself, splanchnic congestion developing secondary to significant reduction in the size of the portal sinusoidal bed, and a certain degree of portal hypertension developing in the postoperative period [28]. These events are the same regardless of the surgical approach. As well, due to inability to perform manual compression of the cut surface during transection, we frequently use the intermittent Pringle maneuver during laparoscopic hepatectomy to limit blood loss during parenchymal transection. For this reason, rates of utilization and overall length of hepatic hilar clamping were significantly greater for LHH than OHH. This, too, might have contributed to the 14% rate of postoperative paralytic ileus following LHH that we observed in our series. Given that return to normal bowel function is one of the key endpoints in some prospective trials aiming to determine the impact of the laparoscopic approach on “functional recovery” following surgery [29–31], this finding is noteworthy and a cause for reflection as to whether such an endpoint is the most appropriate to evaluate the benefits and impact of laparoscopy in the setting of this particular procedure or procedures of a similar nature.

Pulmonary complications are common following liver resection, arising in > 20% of cases in most series [32, 33]. A French multicenter study specifically focused on pulmonary complications following major hepatic resection and detected a high rate for open procedures (41%) that was significantly reduced following laparoscopic procedures (13%) [34]. In this study, pulmonary complications (including pneumonia, pleural effusion, and pulmonary embolism) were detected in 19% of patients following OHH but only 2% of patients following LHH. Actual or perceived pain and other functional limitations associated with open incision and costal retraction in OHH contribute to suboptimal pulmonary hygiene in the postoperative period. Following laparoscopic and even hybrid hemihepatectomy; however, early abdominal wall pain and function are considerably improved, leading to better respiratory and earlier physical activity.

Medbery and colleagues introduced the concept of “value” into the armamentarium of the benefits offered by laparoscopic liver surgery—that quality (e.g., outcomes) can be improved while cutting costs [8]. Unlike the singular and largely nascent endpoints that are used in most surgical trials, value takes into account not only perioperative events but also more remote outcome measures, such as physical recovery, ongoing pain, incisional hernias, disease recurrence, and survival. Given that the outcome benefits that laparoscopy offers may only be compounded over time, LHH appears to offer a value advantage over OHH. Furthermore, the value of LHH is likely to improve further still as surgical expertise increases and upfront costs associated with the operative procedure progressively reduce due to shorter operative times and the ability to perform procedures with a more limited range of surgical instruments and devices. This is especially true for new generations of surgeons, who train directly in laparoscopy in other abdominal interventions (e.g., cholecystectomies, appendectomies, colectomies) and will also train directly in laparoscopic hepatectomies using standardized techniques honed by their predecessors.

The present study does have limitations related to its retrospective nature and the non-randomized distribution of patients. Inverse probability of treatment weighting is a propensity method that does not remove patients but gives them distinct weights based on over- or underrepresentation of certain influential characteristics. Following IPTW adjustments, the results of this study continue to support the raw and unadjusted findings. Nonetheless, even IPTW adjustments cannot achieve the same balanced distribution of unquantified or unidentified variables as in a randomized design [35]. Another limitation is the relatively small sample size, which is partially a consequence of increasing use of parenchymal-sparing surgical techniques, particularly for the resection of metastatic lesions [36]. As well, there is the fact that ten patients in the LHH group were operated using a hybrid approach. These patients belonged to the first 3 years of the inclusion period. It is important to note that all hybrid cases were planned preoperatively and were not unplanned conversions, which have been shown to be associated with greater perioperative morbidity and mortality [37]. Comparing the 35 pure laparoscopic with the ten hybrid hemihepatectomies, both CCI at discharge and 90 days and costs at discharge and 90 days tended to be lower for the hybrid procedures, by and large based on the fact that intraoperative costs for the planned hybrid procedures were lower (7260€ [5723–7988] and 4937€ [3473–6954], respectively, *p* = 0.024).

In summary, while laparoscopic hemihepatectomy remains a complex surgical procedure, it appears to offer considerable benefits over the open approach in terms of morbidity and costs arising postoperatively, not only during index admission but also in subsequent readmissions. These savings in the postoperative period serve to offset the initial increase in operating room costs associated with laparoscopy. In the future, as experience with and results following minimally invasive liver surgery continue to improve, it is plausible that the laparoscopic or other minimally invasive approaches may eventually become the standard-of-care for hemihepatectomy as they already have for other more minor forms of liver resection.
